# Commentary on Andreacchi *et al.*: Policy responses to shifting epidemiological trends in alcohol use in Canada

**DOI:** 10.1111/add.16704

**Published:** 2024-11-04

**Authors:** Carolin Kilian, Charlotte Probst

**Affiliations:** 1 Institute for Mental Health Policy Research, Centre for Addiction and Mental Health (CAMH), Toronto, Ontario, Canada; 2 Center for Interdisciplinary Addiction Research (ZIS), Department of Psychiatry and Psychotherapy, University Medical Center Hamburg-Eppendorf (UKE), Hamburg, Germany; 3 Campbell Family Mental Health Research Institute, Centre for Addiction and Mental Health, Toronto, Ontario, Canada; 4 Department of Psychiatry, University of Toronto, Toronto, Ontario, Canada; 5 Faculty of Medicine, Institute of Medical Science, University of Toronto, Toronto, Ontario, Canada

**Keywords:** Alcohol policy, alcohol use, alcohol-attributable mortality, health inequalities, socio-economic inequalities, socio-economic status

## Abstract

This commentary examines the public health relevance of age, period and cohort effects in heavy episodic drinking in Canada, focusing on socio-economic inequalities and policy implications. It highlights the paradox between observed socio-economic gradients and persistent disparities in alcohol-attributable harm, and contextualizes these findings within the current Canadian policy environment.

In their recent study, Andreacchi and colleagues [1] disentangle epidemiological trends in heavy episodic drinking (HED) by age, period and birth cohort, as well as by sex/gender and socio-economic position (SEP) among Canadian adults. Two indicators of SEP were examined, education and household income, and these yielded two distinct socio-economic patterns.

These patterns reflect a heterogeneous picture that is often observed when examining trends in alcohol use by different indicators of SEP and highlight the importance of acknowledging their differences. The observed income gradient in HED prevalence (i.e. higher prevalences with higher incomes) mirrors the financial resources individuals have available for purchasing alcoholic beverages. Compared to those with low incomes, individuals with high incomes spent a lower proportion of their income on each unit of alcohol, making it more affordable. Education, on the other hand, comprises other aspects potentially underlying drinking decisions, such as drinking opportunities, drinking culture or health literacy, leading to a less distinct socio-economic pattern and additional differences by sex/gender.

In Canada and elsewhere, low-SEP individuals experience considerably higher alcohol-attributable mortality compared to those with high SEP [2]. This has been observed for both education and income measures [3, 4] and linked to HED [5]. It is thus remarkable that Andreacchi and colleagues did not find corresponding gradients in HED prevalence. On the contrary, HED was found to be lowest in the low-income group, while there was no clear gradient for education. To this end, it should be noted that the HED prevalence presented in this study refers to the entire population. Previous studies, looking at current alcohol users only, found an inverse relationship between SEP and HED [6, 7], indicating a more polarized consumption pattern in low-SEP individuals (i.e. higher prevalences of both abstinence and HED). Moreover, measurement limitations are likely to bias the observed socio-economic patterns. We need to acknowledge that surveys are limited in their ability to reach low-SEP and high-risk drinking groups due to self-selection conditional on alcohol use and SEP, as well as sampling frames excluding very specific populations, such as institutionalized individuals [8].

Andreacchi and colleagues further found a marked drop in the HED prevalence among all income and education levels in the youngest birth cohort (1990–2009), with the low-education group having the lowest prevalence [1]. Although it remains inconclusive whether this trend and shift in education patterns will continue in younger birth cohorts, it is important to explore possible drivers, such as a potential loss of status of drinking alcohol among the youngest cohorts, as has been observed in European countries [9]. However, the declines may be short-lived, given Canada’s recent progression towards liberalization of alcohol policies [10].

Due to privatization of alcohol retailing in several provinces [11] and a general trend towards looser pricing regulations [10], Canada’s formerly strict alcohol control framework has become increasingly liberal over the past decades. This erosion of alcohol policies may very well be reflected in increasing alcohol-related harms in the near future. Furthermore, despite declines in HED among men and the youngest birth cohort, this drinking pattern remains prevalent in Canada, with one in 10 women and one in five men engaging in HED in 2021 [1]. There are several alcohol policy options available to address HED and the unequal health burden related to it. Pricing policies in particular are not only highly cost-effective in lowering overall alcohol use, but may also result in greater health benefits in low-SEP groups and high-risk alcohol users, who would be most affected by lower alcohol affordability through higher beverage prices [12]. Moreover, beverage-specific excise duties can target alcohol use in high-risk population by levying higher excise taxes on alcoholic beverages that are either most consumed in these groups or known to be linked to greater health risks. For example, spirits have been found to be preferably consumed in low-SEP and high-risk drinking groups [13] and associated with health harms linked to HED [14].

Against Canada’s progressive alcohol policy liberalization, policy responses to address individuals engaging in HED and bearing a disproportionate alcohol-attributable health burden are required. Detailed epidemiological trend analysis, as conducted by Andreacchi and colleagues, provides important insights to identify relevant population groups and emerging trends.

## Data Availability

N/A.
